# Targeting IL-17A Improves the Dysmotility of the Small Intestine and Alleviates the Injury of the Interstitial Cells of Cajal during Sepsis

**DOI:** 10.1155/2019/1475729

**Published:** 2019-08-18

**Authors:** Jing Li, Pengyu Kong, Chao Chen, Jing Tang, Xiaoming Jin, Jinglong Yan, Yufu Wang

**Affiliations:** ^1^Department of Pathology and Electron Microscopy Center, Faculty of Basic Medical Science, Harbin Medical University, Harbin, China; ^2^Department of Orthopedics, Second Affiliated Hospital, Harbin Medical University, Harbin, China; ^3^Department of Medical Microbiology and Immunology, University of Alberta, Edmonton, Alberta, Canada

## Abstract

Intestinal dysmotility is a frequent complication during sepsis and plays an important role in the development of secondary infections and multiple organ failure. However, the central mechanisms underlying this process have not been well elucidated. Currently, effective therapies are still lacking for the treatment of sepsis-induced intestinal dysmotility. In this study, we found that the activation of IL-17 signaling within the muscularis propria might be associated with dysmotility of the small intestine during polymicrobial sepsis. Furthermore, we demonstrated that targeting IL-17A partially rescued the motility of the small intestine and alleviated interstitial cells of Cajal (ICC) injury during sepsis. The blockade of IL-17A suppressed the dominant sepsis-induced infiltration of M1-polarized macrophages into the muscularis. Additionally, impaired ICC survival may be associated with the oxidative stress injury induced by dominant infiltration of M1-polarized macrophages. Our findings reveal the important role of the IL-17 signaling pathway in the small intestine during sepsis and provide clues for developing a novel therapeutic strategy for treating gastrointestinal dysmotility during sepsis.

## 1. Introduction

Sepsis is one of the major causes of mortality and morbidity after severe trauma, infection, burn injury, or hemorrhage [1]. Intestinal dysmotility is a frequent complication during sepsis and can lead to ileus barrier dysfunction and microbial translocation [2, 3]. Intestinal dysfunction plays an important role in the development of secondary infections and multiple organ failure [4]. Although several pathogenic mechanisms have been proposed to be involved in sepsis-induced intestinal dysfunction, the central mechanisms underlying this process have not been elucidated [5, 6]. Therefore, effective therapies are still lacking for treating sepsis-induced intestinal dysmotility.

The interleukin 17 (IL-17) family of cytokines and functionally related pathways have been identified as key players in the molecular events that occur during inflammatory and autoimmune diseases [7]. IL-17, which is produced by many different cellular sources, initiates the production of other proinflammatory mediators and chemokines, collectively resulting in an influx of neutrophils [8]. The deleterious role of excessive IL-17 production is well established in the pathogenesis of severe inflammatory diseases [9, 10]. The blockade of IL-17A or its pathways has been demonstrated to effectively improve outcomes in such animal models [11]. During intestinal inflammation, the high level of IL-17 plays an important pathogenic role in the gastrointestinal tract [12, 13]. However, the function of IL-17 in the gut is complex. In addition to its role in the pathogenesis of inflammatory processes, IL-17 is also important for the maintenance and protection of epithelial barriers [14].

In this study, we observed significant activation of the IL-17 signaling pathway in the muscularis propria using a murine model of sepsis. We demonstrated that blockade of IL-17A using a neutralizing antibody alleviated the dysmotility of the small intestine. Our findings revealed the important role of the IL-17 signaling pathway in the small intestine during sepsis and suggested that targeting IL-17A might be a promising strategy for treating dysfunction of the small intestine during sepsis.

## 2. Materials and Methods

### 2.1. Animals

Both adult male and female BALB/c mice (6–8 weeks old, 18–22 g) and female BALB/c mouse pups (8–13 days old) were obtained from the Animal Breeding Center of Harbin Medical University (Harbin, China). All animal care and experimental procedures were carried out in accordance with the Guidelines for the Care and Use of Laboratory Animals (National Research Council, 1996, USA) and were approved by the Institutional Animal Care and Use Committee of Harbin Medical University.

In order to induce septic animals with ileus, a cecal ligation and puncture (CLP) sepsis model was performed as previously described [15–17]. CLP was performed to induce peritonitis. Septic mice received intraperitoneal injections of mouse recombinant anti-IL-17A (5 mg/kg of body weight, R&D Systems) 6 h before CLP. Then, anti-IL-17A was administered in a second injection 6 h after CLP. At 24 h or 48 h after CLP, a 5 cm segment of the jejunum beginning 5 cm distal to the ligament of Treitz was dissected. Small intestinal muscularis strips were prepared by pinning freshly isolated intestinal segments in ice-cold PBS and removing the mucosa facing upward [18]. Some muscle strips were “snap-frozen” in liquid nitrogen and stored at -80°C for further studies.

### 2.2. ICC Isolation and Culture

Small intestinal ICCs were isolated according to the previously described method [19].

RAW264.7 macrophages (from the Type Culture Collection of the Chinese Academy of Science, Shanghai, China) were polarized by culturing them with 100 ng/ml lipopolysaccharide (LPS, Sigma, St. Louis, MO) or 10 ng/ml IL-4 for 12 h. After polarization, the RAW264.7 cells were washed and Dulbecco's modified Eagle's medium (DMEM) (HyClone, Logan, USA) was added for 24 h for conditioning. The isolated ICCs were treated for 24 h by replacing 50% of the M199 medium with M1- or M2-derived medium to investigate the effect of polarized macrophages on ICCs.

### 2.3. Electron Microscopy

The ICCs or tissue samples from the muscularis propria of the small intestine were fixed for 2 h with 2.5% glutaraldehyde in PBS (pH 7.2). After being rinsed, the specimens were postfixed in 1% osmium tetroxide for 1 h at 4°C, then dehydrated and embedded in Epon 812. Ultrathin sections were cut at a thickness of 50–70 nm, stained with uranyl acetate and lead citrate, and examined using a HITACHI H-7650 electron microscope.

### 2.4. Immunohistochemistry and Immunofluorescence

For H&E staining, intestinal samples were fixed for 24 h using 4% paraformaldehyde and stained with H&E after dehydration. After the samples were embedded and sliced, morphological changes were observed under a light microscope (Nikon, Tokyo, Japan). The histological damage was evaluated according to previously described criteria [20].

For immunohistochemical analysis, small intestinal tissues (4 *μ*m) were blocked with 5% BSA and incubated with rabbit-derived antibodies specific for CD68 (1 : 200), CD163 (1 : 250), inducible nitric oxide synthase (iNOS, 1 : 200), and arginase 1 (1 : 300) (all primary antibodies were obtained from Abcam, Cambridge, United Kingdom). A biotinylated goat anti-rabbit antibody (1 : 100, Vector Laboratories, Burlingame, CA, USA) was used for visualization. Images were acquired by light microscopy (Nikon, Tokyo, Japan), and staining in the muscular layer was quantified by Image-Pro Plus 7 (Media Cybernetics, Rockville, MD, USA).

For immunofluorescence analysis, the small intestinal tissue was stained using 5% BSA and anti-tyrosine-protein kinase Kit (c-Kit) (1 : 50, Santa Cruz Biotechnology, Dallas, TX, US) at 4°C overnight. The samples were washed in PBS 3 times. Then, the immunoreactivity was detected with a FITC-conjugated secondary antibody (1 : 100, eBioscience, San Diego, CA, US); the nuclei were labeled with DAPI (Fanbo Biochemicals, Beijing, China). Small intestinal samples were analyzed by confocal laser scanning microscopy (Carl Zeiss, Jena, Germany). ICC samples were analyzed by fluorescence microscopy (Nikon, Tokyo, Japan).

Apoptotic cells were investigated by using terminal deoxynucleotidyl transferase- (TdT-) mediated deoxyuridine triphosphate (dUTP) nick-end labeling (TUNEL, Roche, Mannheim, Germany). The cells were finally assessed under a fluorescence microscope.

### 2.5. Myeloperoxidase (MPO) Activity

Myeloperoxidase activity in the small intestinal muscularis tissue was assayed as a measure of leukocyte infiltration, as described previously [21].

### 2.6. Analysis of Myoelectrical Activity

The myoelectrical activity and intraluminal pressure of the intestine were recorded as described previously [19, 22, 23]. The mice were anesthetized with amobarbital sodium. A 1.5 cm midline abdominal incision was made, and one platinum electrode was placed on the muscular layer under the serosa; the proximal electrodes were placed 1 cm beyond the initial point of the jejunum to avoid breaking through the intestinal wall. With the same methods, the other platinum electrode was placed at 2 cm across the length at the beginning of the jejunum. This approach ensured that the electrodes were located along the same axis. The strips were equilibrated for 20 min with a 0.5 g preload. Intestinal contractile activity recordings were then performed. Frequency of contractions was counted for 20 min, and the average number of contractions per minute in each sample was calculated. Amplitude was calculated as area under the curve for 5 min for each sample. The parameters were set as described [19].

### 2.7. RNA Extraction and Microarray Hybridization

Small intestinal muscularis propria tissues from septic mice and control mice were obtained for the microarray analysis. The tissues were immediately frozen in a mixture of isopentane and dry ice and stored at -80°C. RNA was extracted by homogenizing each sample in 1 ml of TRIzol reagent (Invitrogen, Carlsbad, CA, USA). RNA quantity and quality were measured with a NanoDrop ND-1000 spectrophotometer (Wilmington, DE, USA). The A260/A280 ratio between 1.8 and 2.0 is considered acceptable for usable samples. RNA integrity was assessed by standard denaturing agarose gel electrophoresis. Sample labeling and array hybridization were performed according to the Agilent One-Color Microarray-Based Gene Expression Analysis protocol (Agilent Technologies).

### 2.8. Reverse Transcriptase Polymerase Chain Reaction (RT-PCR)

Small intestinal muscularis propria tissues and samples of ICC cells were collected for RT-PCR analysis. RNA was isolated by RNAzol (Invitrogen). cDNA was synthesized from total RNA using the PrimeScript RT reagent Kit (TaKaRa, Dalian, China) according to the manufacturer's instructions. Real-time PCR was performed with SYBR green (Roche, Mannheim, Germany) on a StepOne Real-Time PCR System (Applied Biosystems). Expression data were normalized to *β*-actin mRNA expression.

### 2.9. Analysis of NF-*κ*B and MAPK Signaling Activation

The activation of NF-*κ*B and MAPK signaling was measured by DNA-binding activity with TransAM Transcription Factor ELISA Kits (Active Motif, Japan). Nuclear extracts were collected using a Nuclear Extract Kit (Active Motif). Proteins were quantified using the BCA method and subjected to an ELISA-based TransFactor assay.

### 2.10. ELISA

The expression levels of IL-6, IL-12, and IL-10 were determined in the harvested samples (all layers of the small intestine) using the corresponding ELISA kits (Elabscience, Wuhan, China). The absorbance was read at 450 nm by an automated ELISA plate reader (BioTek, Winooski, VT, USA).

### 2.11. Statistical Analysis

The results are expressed as the means ± SD. The independent samples *t*-test or the Mann-Whitney *U* test was used to assess the differences between two groups. Differences among more than two groups were evaluated by using one-way analysis of variance (ANOVA) or the Kruskal-Wallis test. *P* values below 0.05 were accepted as significant. Statistical analysis was performed using SPSS 16.0 software (SPSS, Chicago, IL, USA).

## 3. Results

### 3.1. Sepsis Activated the IL-17 Signaling Pathway within the Muscularis Propria

**Figure 1:**
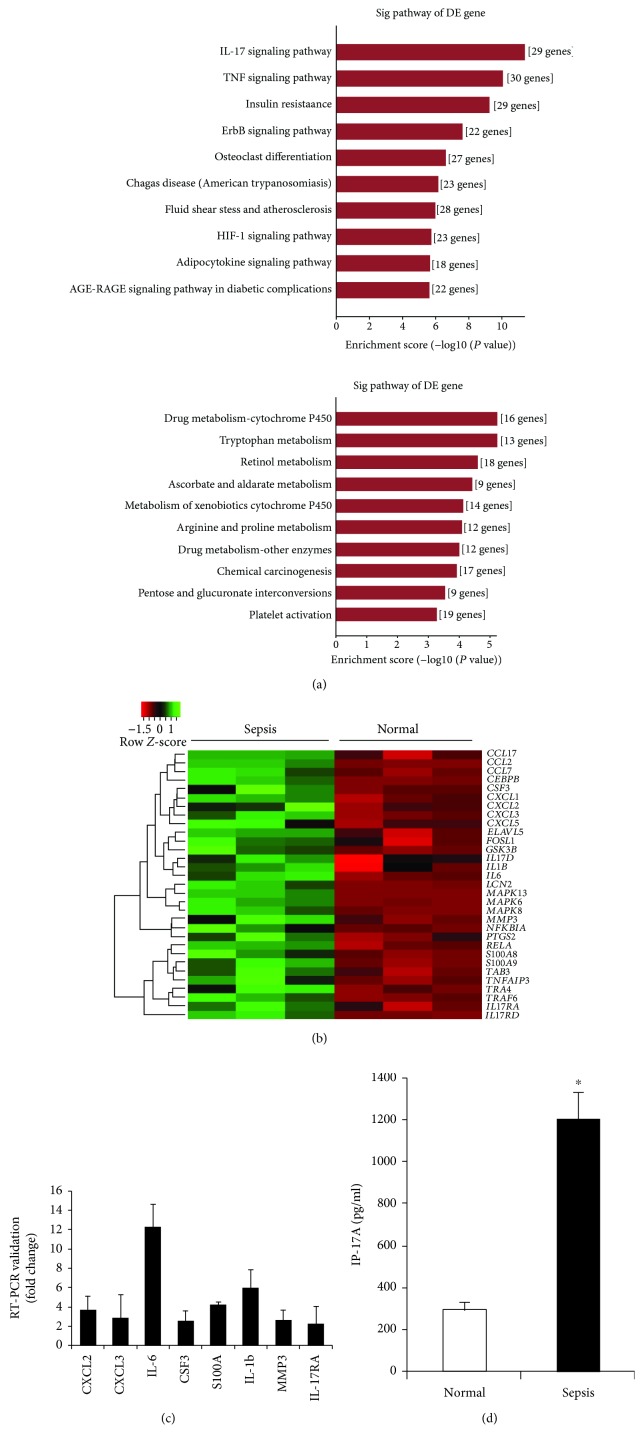
IL-17 signaling pathway in the small intestinal muscularis propria in normal mice and septic mice. Microarray-based genome-wide gene expression profiles were investigated 48 h after CLP. (a) The results showed the top ten enrichment score (-log10 (*P* value)) values of the upregulated and the downregulated genes and significant enrichment pathways. (b) The significantly upregulated genes related to the IL-17 signaling pathway (heat map) in septic mice. The higher the enrichment score is, the more significant the pathway, *n* = 3 in each group. (c) Real-time PCR analysis for the validation of selected gene expression patterns. (d) Comparison of the protein expression of IL-17A in the muscularis propria between normal mice and septic mice (data are shown as the mean ± SD, *n* = 3 to 4, ^∗^*P* < 0.05).

To investigate the biological mechanism that underlies impaired motility of the small intestine during sepsis, gene expression profiles within the muscularis propria were compared between normal and septic mice. Although many genes were differentially expressed ([Supplementary-material supplementary-material-1]), the strongest association was found between septic mice and the IL-17 signaling pathway. The pathways with the top ten enrichment scores of the upregulated and the downregulated genes are shown in Figures 1(a) and 1(b). In upregulated genes, the pathway with the most significant changes was the IL-17 signaling pathway. Significant differences in the expression of IL-17 pathway-related genes are shown in Figure 1(c). Several genes were selected for validation by real-time PCR (Figure 1(b)). Furthermore, we evaluated the IL-17A level in the muscular layer of the small intestine and found that the expression of the IL-17A protein was significantly increased in septic mice compared to control mice (Figure 1(d)). These data suggested that the activation of IL-17 signaling in the muscular layer might be associated with dysmotility of the small intestine during sepsis.

### 3.2. Blockade of IL-17A Improved Dysmotility of the Small Intestine during Sepsis

**Figure 2:**
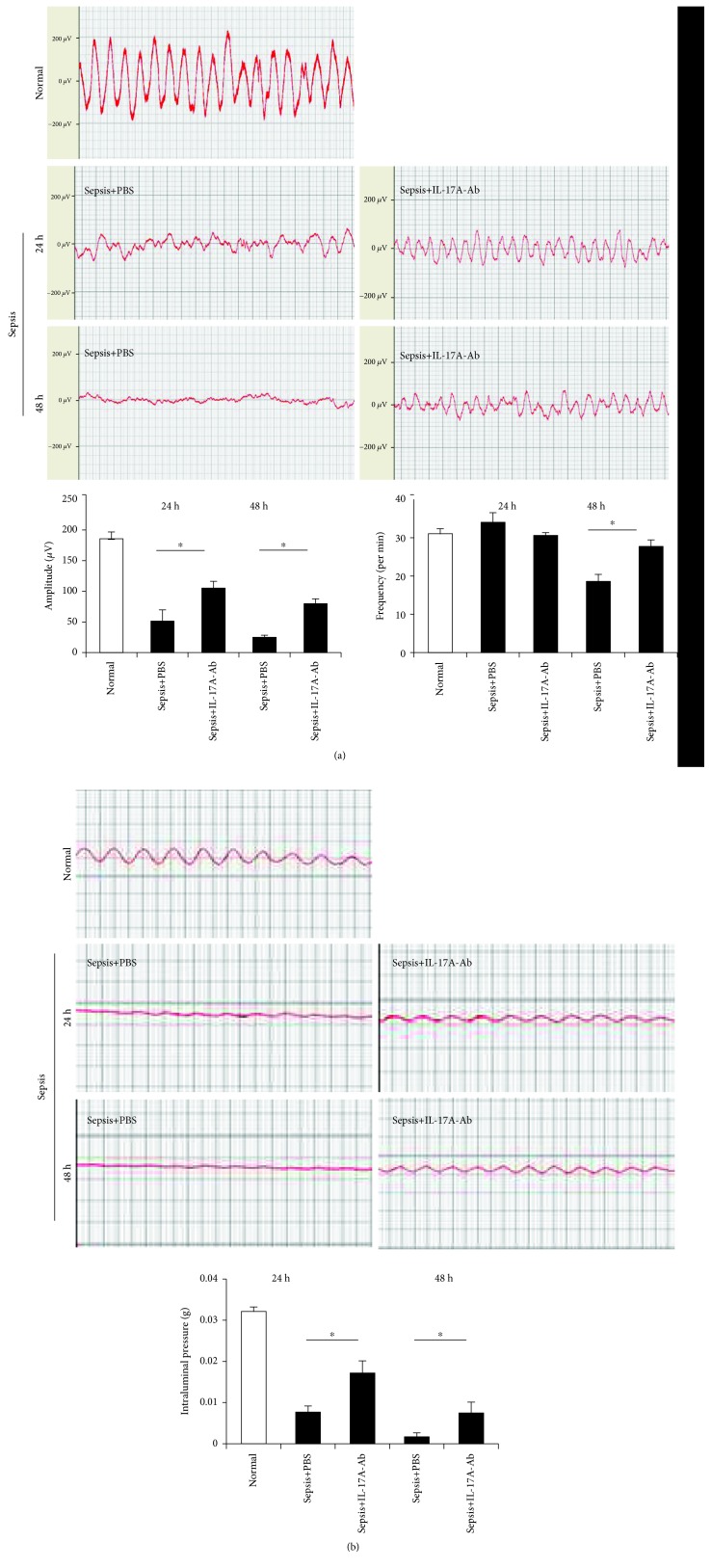
Motility of the small intestine in normal mice, septic mice treated with PBS, and septic mice treated with IL-17A-Ab. (a) Analysis of intestinal myoelectrical activity. The amplitude and frequency were compared among the groups 24 h and 48 h after CLP. (b) Analysis of intraluminal pressure. The intraluminal pressure of the small intestine was compared among the groups 24 h and 48 h after CLP (data are shown as the mean ± SD, *n* = 5 to 6, ^∗^*P* < 0.05).

The results of the pathway analysis prompted us to investigate the effect of blocking IL-17 signaling on the motility of the small intestine. Therefore, the myoelectrical activity of the small intestine was compared between septic mice and septic mice that were treated with an anti-IL-17A antibody (IL-17A-Ab). As shown in Figure 2(a), the amplitude of the slow wave was significantly reduced in septic mice compared to that of normal mice. We also found that the frequency of the waveform was significantly changed 48 h after CLP. However, the myoelectrical activity of the small intestine in septic mice was partially recovered by IL-7A-Ab treatment, as demonstrated by the increased frequency and amplitude. Additionally, neutralization of IL-17A improved the intraluminal pressure of the small intestine during sepsis (Figure 2(b)).

### 3.3. Blockade of IL-17A Alleviated Sepsis-Induced ICC Damage

**Figure 3:**
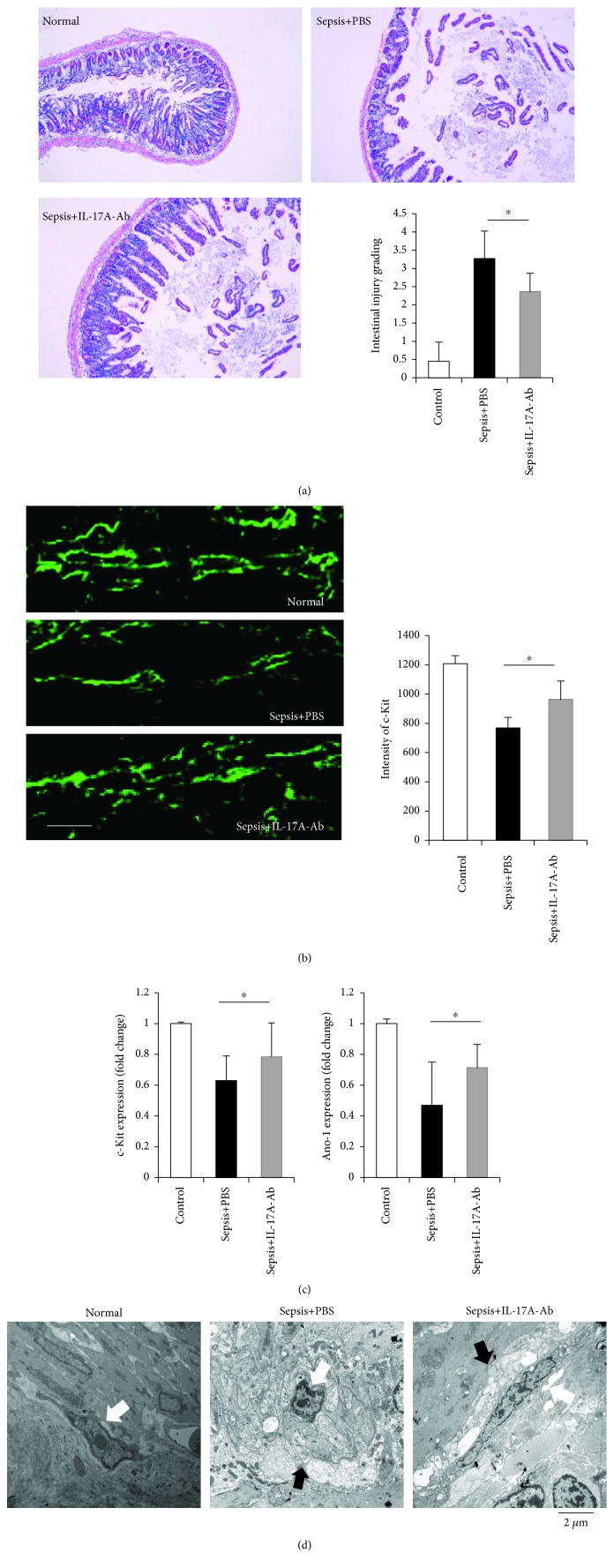
Histological analyses of the small intestine and changes in ICCs in normal mice, septic mice treated with PBS, and septic mice treated with IL-17A-Ab. (a) Small intestine samples were obtained 48 h after CLP. The histologic scoring of hematoxylin and eosin (H&E) staining was assessed by intestinal injury grading. Scale bar: 0.2 mm. (b) Immunohistochemical staining for c-Kit was performed to analyze ICC networks. The immunofluorescence intensity of c-Kit was assessed. Scale bar: 50 *μ*m. (c) RT-PCR analysis of the c-Kit and Ano-1 expression in the small intestinal muscularis propria. (d) Ultrastructural changes in ICC morphology were examined by TEM analysis (10,000x magnification). The damage to ICCs (white) and swelling of the nerve filament (black) are indicated by arrows (data are shown as the mean ± SD, *n* = 5 to 7, ^∗^*P* < 0.05).

Through morphological analysis, we found that the neutralization of IL-17A significantly decreased the intestinal injury scores in septic mice (Figure 3(a)). Confocal images suggested that septic mice exhibit a decreased number of ICCs and a decrease in the ICC network compared to those of normal mice (Figure 3(b)). However, a partially recovered intensity of the c-Kit expression was observed in IL-17A-Ab-treated mice, suggesting the presence of an increased number of ICCs (Figure 3(c)). In addition, treatment targeting IL-17A also elevated the low mRNA expression levels of c-Kit and Ano-1, which are specific biomarkers that are expressed in ICCs and induced by sepsis. The protective effect of IL-17A-Ab on injured ICCs was further confirmed by transmission electron microscopy (TEM) (Figure 3(d)). The highly condensed nuclei, swollen mitochondria, and significantly shortened processes were partially recovered by IL-17A-Ab treatment. Notably, the swelling of nerve fibers, which suggests neuronal injury in the inflamed small intestine, was also alleviated after the blockade of IL-17A.

### 3.4. Blockade of IL-17A Suppressed Inflammation within the Muscularis Propria

**Figure 4:**
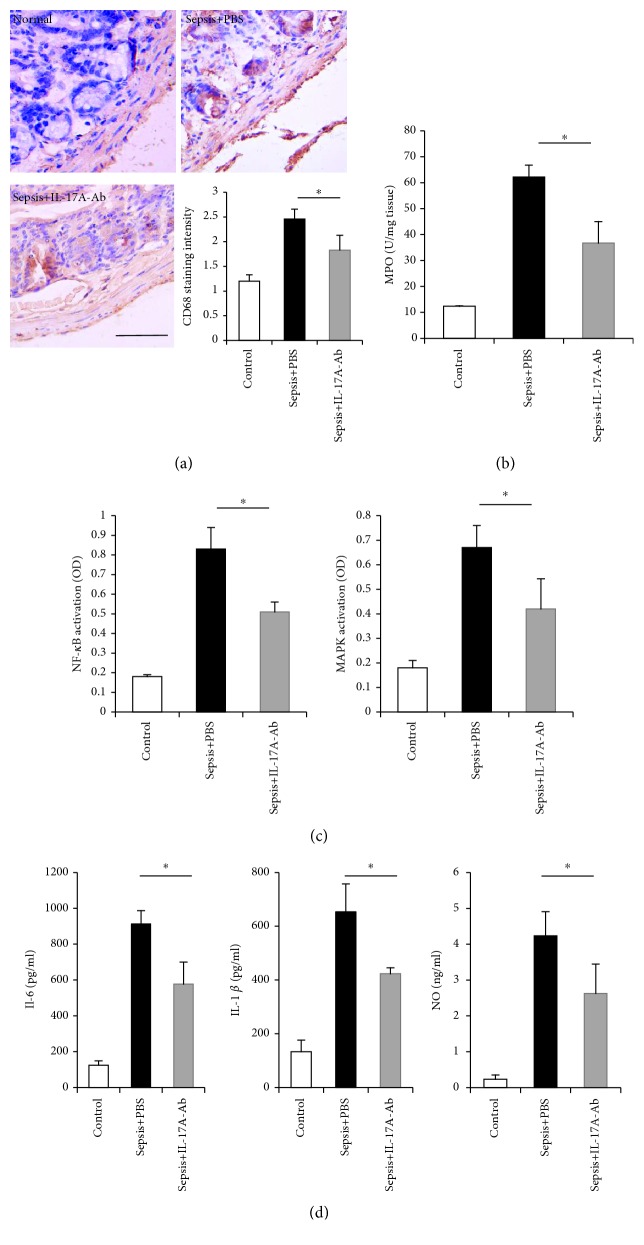
The inflammatory response within the small intestinal muscularis propria in septic mice treated with PBS and septic mice treated with IL-17A-Ab. Samples were obtained 48 h after CLP. (a) Representative pictures of the small intestine stained with CD68 for macrophages. Quantification of the macrophages infiltrating the muscularis propria was compared. Scale bar: 100 *μ*m. (b) The quantification of MPO, a marker of neutrophil activation, is presented among each group. (c) The activation of NF-*κ*B and MAPK signaling was examined by DNA-binding activity. (d) The expression of IL-6, IL-1*β*, and NO was measured by ELISA. NF-*κ*B: nuclear factor kappa B; MAPK: mitogen-activated protein kinase; MPO: myeloperoxidase (data are shown as the mean ± SD, *n* = 5 to 7, ^∗^*P* < 0.05).

To further delineate the mechanism by which blocking IL-17A improved the motility of the small intestine, we analyzed the inflammatory microenvironment in the muscularis propria. In the muscular layer of the small intestine, blocking IL-17A ameliorated sepsis-induced inflammation, as evidenced by a decreased number of CD68^+^ macrophages and decreased levels of MPO activity (Figures 4(a) and 4(b)). Moreover, sepsis caused significant activation of NF-*κ*B and MAPK signaling, which was suppressed by treatment with IL-17A-Ab (Figure 4(c)). As expected, IL-17A-Ab administration also strongly inhibited the production of proinflammatory cytokines, including IL-6, IL-1*β*, and NO, within the muscularis propria (Figure 4(d)).

### 3.5. ICC Injury Is Associated with M1 Macrophage-Mediated Oxidative Stress

**Figure 5:**
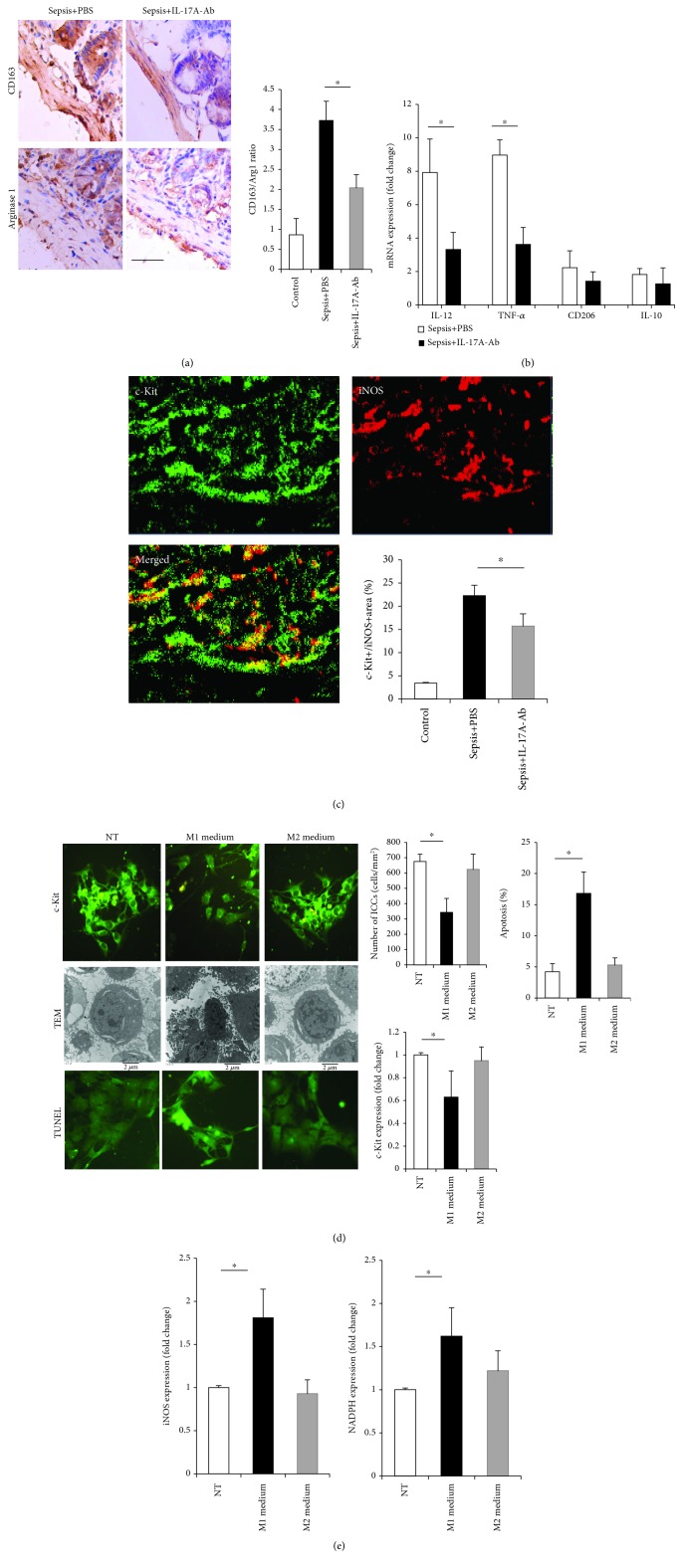
ICC injury is associated with M1 macrophage-mediated oxidative stress. Samples were obtained 48 h after CLP. (a) Altered M1 and M2 macrophage infiltration in the muscularis propria. Representative images of M1 (CD163) staining and M2 (arginase 1) staining are shown. The ratio of M1/M2 macrophages was further analyzed in each group. Scale bar: 100 *μ*m. (b) RT-PCR analysis of the expression of marker genes related to M1 and M2 macrophages in the muscularis propria. There was a significant reduction of M1 macrophage marker genes. (c) Double-immunostaining of c-Kit (green) and iNOS (red) in the muscularis propria was performed, and the c-Kit-positive and iNOS-positive areas were analyzed. Representative images of c-Kit and iNOS staining from septic mice are shown. Scale bar: 20 *μ*m. (d) The pathological changes in ICCs after treatment with M1- or M2-derived medium for 24 h. ICCs were detected by c-Kit immunocytochemical staining (200x magnification), TUNEL (400x magnification), and TEM (10,000x magnification). The number of ICCs, the expression of c-Kit, and the percentage of apoptotic cells were also analyzed. (e) Expression levels of iNOS and NADPH in ICCs treated with M1- or M2-derived medium for 24 h. NT: no treatment; Arg1: arginase 1; NADPH: nicotinamide adenine dinucleotide phosphate (data are shown as the mean ± SD, *n* = 5 to 7, ^∗^*P* < 0.05).

Based on the observed inhibitory effect of blocking IL-17A on muscularis inflammation, we sought to investigate the factors that alleviated ICC damage. Since IL-17A-Ab treatment did not alter the number of ICCs or the level of c-Kit expression *in vitro* ([Supplementary-material supplementary-material-1]), we focused on macrophage polarization. IL-17A significantly decreased both CD163 and arginase 1 expression compared with that of septic mice, whereas the CD163/arginase 1 ratios were lower in the IL-17A-Ab-treated septic mice than in the septic mice (Figure 5(a)). To corroborate the histological findings, the expression of M1 markers (IL-12 and TNF-*α*) and M2 markers (CD206 and IL-10) was quantified in the muscular layer tissues using RT-PCR. The increased expression of IL-12 and TNF-*α* in septic mice was significantly inhibited after IL-17A administration, while the expression of CD206 and IL-10 was not significantly inhibited (Figure 5(b)). These data suggested that the blockade of IL-17A suppressed the dominant infiltration of M1-polarized macrophages and altered the ratio of M1/M2 macrophages. In addition, we found that sepsis led to a larger iNOS^+^ and c-Kit^+^ area in the muscularis, suggesting that ICC injury is related to iNOS-mediated oxidative stress (Figure 5(c)).

Next, we wondered whether M1 macrophages are associated with ICC injury. We demonstrated that treatment of ICCs with M1 macrophage-derived medium led to significant decreases in ICC networks and numbers, as well as an increase in the number of apoptotic cells (Figure 5(d)). Ultrastructural examination further confirmed the damage to ICCs, including condensed nuclei, reduced volume, and condensed, shortened, or reduced processes. M1 macrophage-derived medium also affected the number of ICCs and the mRNA expression of c-Kit and led to more apoptotic ICCs than observed in the nontreatment (NT) group or the M2 macrophage-derived medium group (Figure 5(d)). However, M2 macrophage-derived medium did not show a significant influence on ICC morphology or the expression of c-Kit. Furthermore, IL-17A, which can be expressed by M1 macrophage, did not show a significant influence on the number of ICCs and c-Kit expression (see [Supplementary-material supplementary-material-1]). Through an *in vitro* experiment, we demonstrated that M1 macrophage-derived medium significantly increased the expression of oxidative stress-related genes, including iNOS and NADPH (Figure 5(e)). These data suggested that M1 macrophages might be an important cause of ICC damage and that this effect is related to iNOS-mediated oxidative stress.

## 4. Discussion

In sepsis, the intestine is not only a source of bacteremia but also an important target of bacterial products with major functional consequences to intestinal motility and the generation of cytokines, which participate in the development of multiple organ failure [15]. There is emerging evidence that IL-17 may play a distinct role in regulating the immune response and represent a potential therapeutic target in inflammatory diseases. In this study, we demonstrated that IL-17 signaling within the muscularis propria might be associated with dysmotility of the small intestine during sepsis. Neutralization of IL-17A inhibited inflammation within the muscular layer and partially recovered the motility of the small intestine during sepsis. We also demonstrated that anti-IL-17A treatment ameliorated ICC injury by suppressing M1 macrophage activation.

As IL-17A plays key regulatory roles in host defense and inflammatory processes, there is still a controversy concerning its proinflammatory and anti-inflammatory functions. IL-17A is associated with the development of various arthritic, allergic, and autoimmune disorders [24]. Higher IL-17A expression was found in the gut during intestinal inflammation, which may serve a protective function in bacterial clearance, inflammation control, and epithelial barrier maintenance [25, 26]. In addition, IL-17 has been linked to the severity of inflammation in tissues. In septic animal models, a neutralizing antibody against IL-17A exerted protective effects by reducing bacteremia, lung injury, and abscess formation [9, 27, 28]. The upregulated levels of IL-17A during sepsis might be harmful to the small intestine [29]. Our data demonstrated that the transcript level of IL-17A was not significantly changed within the muscularis propria, suggesting that the high level of IL-17A might partially originate from elevated systemic levels of IL-17A. Moreover, the recruited Th17 cells in the mucosal lamina propria might be another source of upregulated IL-17A levels in the muscularis propria.

In the current study, the significant alteration of IL-17 signaling pathways in the muscle layer in septic mice led us to hypothesize that blocking IL-17A could contribute to improving the dysmotility of the small intestine induced by sepsis. We found that the myoelectrical activity and intraluminal pressure of the small intestine were partially recovered in septic mice after treatment with IL-17A-Ab, suggesting that the blockade of IL-17A may provide protection to the muscularis propria. The function of gastrointestinal motility is mainly regulated by ICCs, smooth muscle cells (SMCs), and enteric neurons. Among all the cell types associated with motility, ICCs play a core role. They function as a pacemaker and generate spontaneous electrical activity (slow wave), which mediates input from the enteric motor nervous system to smooth muscle, resulting in rhythmic contractions [30, 31]. The decreased number of ICCs and the weakened ICC network may result in abnormal intestinal motility and delayed transit [5, 32, 33]. The dysmotility of the small intestine during sepsis is an important cause of barrier dysfunction or microbial translocation and, correspondingly, increased mortality [6]. Through general morphological and ultrastructural analysis, we found that the blockade of IL-17A not only prevented the survival of ICCs and weakened the network of ICCs but also reduced the swelling of nerve filaments.

In the inflamed muscularis propria, a shift in the proportion of M1/M2 macrophages after blocking IL-17A might be attributed to multiple factors, including the modulation of polarization, impaired recruitment, and altered survival of specific populations. Previous studies have demonstrated that IL-17A can stimulate the secretion of proinflammatory cytokines from macrophages. IL-17A inhibition or deficiency may significantly affect macrophage polarization and accumulation in the lesion area [34–36]. We demonstrated that ICC injury could be caused by M1-polarized macrophages. In the normal intestine, macrophage phenotypes differ depending on their location, with macrophages in the muscularis propria layer preferentially assuming an anti-inflammatory M2 phenotype, while macrophages in the lamina propria display a proinflammatory M1 phenotype [37, 38]. Under physiological conditions, macrophages play a key role in regulating the function of ICCs and the immune microenvironment [39, 40]. However, sepsis disrupts the M1/M2 balance in the muscularis propria, which is characterized by an M1 macrophage-dominated inflammatory response.

Previous studies illustrated that individual cytokines may have a limited effect on the function of ICCs [41]. Our findings also indicated that ICC may have low sensitivity to IL-17A. Therefore, the damaged ICCs observed during sepsis may be the result of synergistic action by multiple inflammatory cytokines. Among those inflammatory mediators, NO produced by iNOS may be a main factor in ICC injury and intestinal dysmotility. NO has deleterious effects on the function and network of ICCs through mechanisms that depend on oxidative stress [41, 42]. Moreover, tumor necrosis factor alpha (TNF-*α*) is involved in M1-macrophage-induced ICC injury [43]. In this study, the upregulation of iNOS and NADPH expression suggested that ICC injury is associated with increased oxidative stress induced by M1 macrophages. The NF-*κ*B and MAPK pathways have also been implicated in the inflammation-mediated dysfunction of ICCs [44].

## 5. Conclusion

In summary, targeting IL-17A was an effective strategy for controlling the dysmotility of the small intestine during sepsis. We revealed that the IL-17 signaling pathway might be involved in the development of dysmotility of the small intestine during sepsis. Within the muscularis propria, impaired ICC survival and function may be associated with the dominant infiltration of M1-polarized macrophages. The findings of this study provide a clue for developing a novel therapeutic strategy for treating gastrointestinal dysmotility in sepsis.

## Data Availability

The data used to support the findings of this study are available from the corresponding author upon request.

## Supplementary Materials

Supplementary 1 Supplementay Table: Different gene expression profile data between normal and septic mice.

Supplementary 2 Supplementay Figure: The effect of IL-17A on ICCs. (A) Representative images of ICCs treated with IL-17A at concentrations of 50 ng/ml for 24 h. (B) There was no significant difference between NT ICCs and IL-17A-treated ICCs in the number of ICCs and the expression of c-Kit. The evaluation of ICCs was conducted at 24 h after exposure to IL-17A at concentrations of 10 ng or 50 ng/ml for 24 h. NT: no treatment.
