# Supplementary material for: Targeting IL-17A Improves the Dysmotility of the Small Intestine and Alleviates the Injury of the Interstitial Cells of Cajal during Sepsis
Source: Oxid Med Cell Longev. 2019 Aug 18;2019:1475729. doi: 10.1155/2019/1475729 (PMC6721283; doi:10.1155/2019/1475729)
Supplement: Supplementary 2 — Supplementay Figure: The effect of IL-17A on ICCs. (A) Representative images of ICCs treated with IL-17A at concentrations of 50 ng/ml for 24 h. (B) There was no significant difference between NT ICCs and IL-17A-treated ICCs in the number of ICCs and the expression of c-Kit. The evaluation of ICCs was conducted at 24 h after exposure to IL-17A at concentrations of 10 ng or 50 ng/ml for 24 h. NT: no treatment. [file 1475729.f1.docx]

**Targeting IL-17A signaling improves dysmotility of the small intestine and** **alleviates interstitial Cajal cell injury during sepsis**

Jing Li^*^, Pengyu Kong^*^, Chao Chen, Jing Tang, Xiaoming Jin, Jinglong Yan, Yufu Wang

**Supplementary figure**


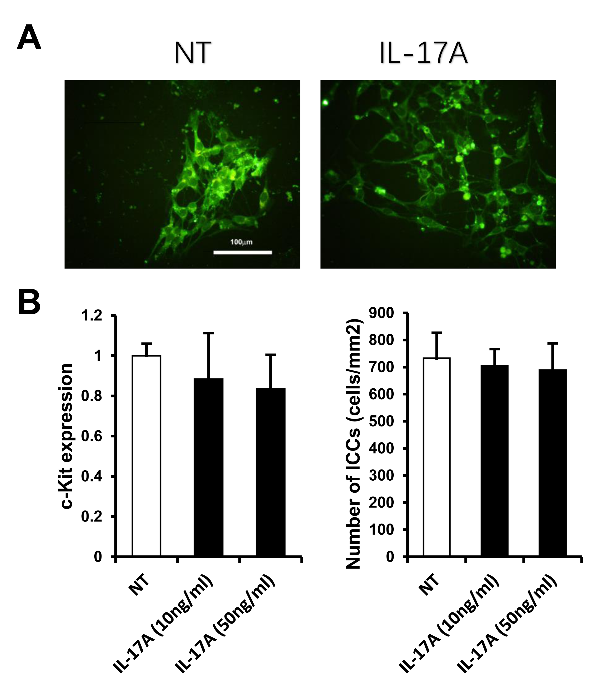


**Supplementary Figure** The effect of IL-17A on ICCs. A. Representative images of ICCs treated with IL-17A at concentrations of 50 ng/ml for 24 h. B. There was no significant difference between NT ICCs and IL-17A treated ICCs in number of ICCs and the expression of c-Kit. The evaluation of ICCs was conducted at 24h after exposure to IL-17A at concentrations of 10ng or 50 ng/ml for 24 h. NT, no treatment.
